# Digital Activity Frequency and Life Satisfaction Among Older Adults: Indirect Associations Through eHealth Literacy and Attitudes Toward Aging

**DOI:** 10.3390/healthcare14213816

**Published:** 2026-11-09

**Authors:** Yinyinzi Yang, Xiaobo Xu, Cuiping Zhou, Weiguo Pang

**Affiliations:** 1 Shanghai Citizen Lifelong Learning Monitoring and Research Center, Shanghai Open University, 288 Guoshun Road, Yangpu District, Shanghai 200433, China; yyyz@sou.edu.cn (Y.Y.);; 2 School of Psychology, Shanghai Normal University, No. 100 Guilin Road, Xuhui District, Shanghai 200234, China; xbxu@shnu.edu.cn; 3 School of Psychology and Cognitive Science, East China Normal University, 3663 North Zhongshan Road, Shanghai 200062, China

**Keywords:** older adults, digital activity frequency, eHealth literacy, attitudes toward aging, life satisfaction

## Abstract

**Background/Objectives:** Digital participation is increasingly relevant to well-being in later life. This study examined the association between digital activity frequency and life satisfaction and indirect associations through perceived eHealth literacy and three dimensions of attitudes toward aging among older adults. **Methods:** In a cross-sectional survey, 604 Chinese adults aged 60 years or older completed questionnaires assessing digital activity frequency, eHealth literacy, psychosocial loss, physical change, psychological growth, and life satisfaction. A multiple mediation model was estimated with adjustment for gender, education, age category, living arrangement, and community location. Indirect associations were evaluated using bootstrap confidence intervals. **Results:** More frequent digital activity was associated with greater life satisfaction (total association: β = 0.518). Positive indirect associations were found through eHealth literacy alone (estimate = 0.082) and through eHealth literacy followed by either physical change (estimate = 0.060) or psychological growth (estimate = 0.044). Indirect associations through physical change alone and psychological growth alone were also observed, but neither was statistically supported in either sensitivity analysis. Neither indirect pathway involving psychosocial loss was significant. The direct association remained positive after inclusion of the mediators (β = 0.286). **Conclusions:** These findings highlight perceived eHealth literacy and appraisals of physical change and psychological growth as potential links between digital activity frequency and life satisfaction among older adults. They may inform the design and evaluation of support programs that address both eHealth literacy and specific aging-related appraisals in efforts to promote well-being in later life.

## 1. Introduction

Life satisfaction is the cognitive evaluation of one’s life as a whole and a central component of subjective well-being [1]. In later life, life satisfaction is associated with physical health, health behaviors, and psychosocial well-being, making it relevant to person-centered approaches to healthy aging [2,3]. Digital technologies are becoming increasingly integrated into older adults’ everyday lives. Activities such as learning, work-related tasks, entertainment, and social interaction now routinely involve internet-based services [4,5]. Against this background, the frequency of older adults’ everyday digital activities is increasingly relevant to understanding life satisfaction in later life.

Digital activity frequency refers to how regularly older adults engage in routine online learning, work-related activities, entertainment, and social interaction. As one aspect of digital participation, digital activity frequency captures how often older adults engage in these four online activities and is conceptually distinct from initial access to or adoption of digital technologies [4]. Regular participation may provide repeated opportunities to obtain information, maintain social ties, continue learning, and pursue enjoyable or meaningful activities. Previous studies have examined the association between internet use and life satisfaction among older adults. In a longitudinal study conducted in England, infrequent internet use predicted a deterioration in life satisfaction compared with daily use [6]. Among Chinese older adults, internet use was positively associated with life satisfaction, with offline social interaction statistically accounting for part of this association [7]. Other studies have reported variation in the associations of different online activities with life satisfaction [8]. Collectively, this evidence indicates that internet use is relevant to life satisfaction and that its association may operate through different pathways. However, participation in online activities does not necessarily correspond to the skills needed to use digital resources effectively [5]. This distinction raises the question of whether skills in specific domains of everyday life may help explain the association between routine digital activity frequency and life satisfaction.

### 1.1. Indirect Association Through eHealth Literacy

One particularly relevant domain of digital participation in later life is health. Older adults can use the internet to seek health information, communicate with health professionals, access health services, and support health-related decision-making and management [9]. The literacy involved in these activities is commonly conceptualized as eHealth literacy. Norman and Skinner [10] defined eHealth literacy as the ability to locate, understand, evaluate, and use health information obtained from electronic sources to address health-related needs. Although conceptually distinct from digital activity frequency, eHealth literacy draws on computer, information, media, and health literacies. More frequent engagement in these routine online activities may provide experience with online interfaces, navigation, information searches, and the evaluation of digital content, which may be transferable to health-related digital tasks. Cross-sectional studies have found that the frequency and breadth of internet use and engagement in online health-information practices are associated with higher eHealth literacy among older adults [11,12,13,14]. Longitudinal evidence further indicates that longer internet-use histories and more frequent health-related internet use are associated with higher eHealth literacy over time [15].

eHealth literacy may be related to life satisfaction through its relevance to older adults’ pursuit of health-related goals. Health Empowerment Theory (HET) emphasizes the recognition and mobilization of personal and social-contextual resources for purposeful participation in attaining health goals and promoting well-being [16]. From this perspective, perceived eHealth literacy can be understood as a personal resource: confidence in finding, evaluating, and using online health information may support older adults’ participation in health-related decisions and everyday health management. Higher eHealth literacy has been associated with health-promoting behavior and health-related quality of life among older adults, with health-promoting behavior statistically accounting for part of this association [17]. Longitudinal evidence further indicates that eHealth literacy predicts subsequent improvements in health-promoting lifestyles [18]. A recent study also found a significant indirect association between general digital literacy and quality of life through eHealth literacy [19]. More directly related to the present outcome, Wang et al. [20] reported a positive total association between eHealth literacy and life satisfaction.

**H1.**  *Digital activity frequency will show a positive indirect association with life satisfaction through eHealth literacy.*

### 1.2. eHealth Literacy, Attitudes Toward Aging, and Life Satisfaction

eHealth literacy may also be associated with life satisfaction through how older adults view their own aging. Attitudes toward aging encompass evaluations of the changes and experiences associated with growing older [21,22]. Stereotype Embodiment Theory (SET) proposes that age-related beliefs acquired throughout life can become part of individuals’ views of themselves as they grow older, with implications for their experiences and well-being [23]. These beliefs may shape whether age-related difficulties are interpreted as inevitable decline or as challenges that leave room for adaptation. Perceived eHealth literacy may be relevant to such evaluations. Older adults who feel able to find, evaluate, and use health information may perceive more options for responding to health-related changes, rather than viewing these changes solely as consequences of aging that are beyond their control. Such perceptions may be associated with more favorable evaluations of their own aging and, in turn, their lives as a whole. Empirically, more positive attitudes toward aging have been associated with greater life satisfaction [24]. Wang et al. [20] also reported an indirect association between eHealth literacy and life satisfaction through attitudes toward own aging, with self-efficacy included as a parallel mediator. These considerations provide a rationale for examining serial indirect associations through eHealth literacy followed by attitudes toward aging.

From a lifespan-developmental perspective, attitudes toward aging can be understood as multidimensional and multidirectional: evaluations may differ across areas of life, and perceived aging-related gains and losses may coexist [21,25]. Consistent with this perspective, Laidlaw et al. [22] conceptualized attitudes toward aging in terms of three dimensions: psychosocial loss, physical change, and psychological growth. These dimensions concern perceived psychosocial losses associated with aging, evaluations of physical aging, and perceived gains associated with growing older, respectively. Older adults may evaluate different aspects of aging differently: they may perceive psychological growth while also experiencing psychosocial loss or viewing physical aging less favorably. Combining these evaluations into an overall score may obscure their different associations with eHealth literacy and life satisfaction. Examining the three dimensions separately therefore allows the proposed indirect associations to be assessed for each type of aging-related appraisal.

Psychosocial loss reflects perceptions of aging as involving losses in emotional and social life, including loneliness, exclusion, and dependence [22]. More frequent engagement in online activities may be relevant to these perceptions by providing opportunities to maintain social connections and manage everyday needs. In a three-wave longitudinal study, social internet use was associated with greater well-being through lower loneliness and greater social engagement [26]. Perceived eHealth literacy may be relevant to the dependence-related aspect of psychosocial loss. Feeling able to understand and evaluate health information may help older adults participate in decisions about their own care and perceive themselves as capable of addressing health-related needs. A systematic review linked higher eHealth literacy to better health knowledge, decision-making, and self-care behaviors among older adults [27], supporting the possibility that this resource may be associated with a greater sense of independence and a less negative view of aging as increasing dependence. Lower perceived psychosocial loss may, in turn, be relevant to life satisfaction: Chen et al. [28] found that greater psychosocial loss was associated with lower life satisfaction and more depression and loneliness among Chinese older adults.

Physical change reflects evaluations of physical aging, including perceived health and energy. It captures how individuals view their physical aging rather than an objective assessment of disease or functional status. Feeling able to understand and evaluate health information may help older adults view bodily changes as challenges they can respond to, rather than solely as inevitable consequences of aging. A cross-sectional study found that higher eHealth literacy was associated with greater engagement in health-promoting behaviors among older adults [17], and a three-wave longitudinal study found that eHealth literacy predicted subsequent improvements in health-promoting lifestyles [18]. These findings support the relevance of eHealth literacy to everyday health management. Feeling better equipped to manage health-related demands may also be associated with a more favorable appraisal of physical aging. The relevance of this appraisal to later-life outcomes is further supported by a study of 4593 adults from 20 countries, in which physical change showed the strongest indirect association between health satisfaction and quality of life among the three dimensions of attitudes toward aging [29].

Psychological growth reflects perceived gains associated with aging, including wisdom and the value of accumulated experience [22]. eHealth literacy involves finding, evaluating, and applying health information in everyday decisions [9,10]. Feeling capable of learning about health and combining new information with accumulated experience may reinforce older adults’ recognition that they can continue to develop their knowledge and judgment. Applying this knowledge to their own needs or sharing it with others may also affirm the continuing value of their experience, providing a rationale for expecting an association between perceived eHealth literacy and psychological growth. Related evidence links digital engagement to aging-related appraisals. Köttl et al. [30] found that greater engagement with everyday information and communication technologies predicted more positive self-perceptions of aging in the domain of personal competence three years later. Internet use has also been associated with more positive attitudes toward aging among Chinese older adults [31]. Psychological growth may, in turn, be relevant to life satisfaction by helping older adults recognize the continuing value of their experience and view later life as offering possibilities for development and contribution. Such appraisals may support a more favorable evaluation of life as a whole. Consistent with this reasoning, psychological growth has been positively associated with global quality of life [29], and more positive attitudes toward aging have been associated with greater life satisfaction [24]. A systematic review also supports the broader association between positive self-perceptions of aging and quality of life [32].

### 1.3. The Present Study

The present study examines the association between routine digital activity frequency and life satisfaction among Chinese older adults, with a focus on indirect associations through eHealth literacy and attitudes toward aging. Psychosocial loss, physical change, and psychological growth are examined separately to distinguish perceived aging-related losses from favorable appraisals of physical aging and perceived psychological growth, which may relate differently to digital activity frequency, eHealth literacy, and life satisfaction. Specifically, the study examines indirect associations through eHealth literacy alone and serial indirect associations through eHealth literacy followed by each aging-attitude dimension. Examining these pathways together allows a more differentiated assessment of which aging-related appraisals are involved in the association between digital activity frequency and life satisfaction.

**H2.**  *Digital activity frequency will show positive serial indirect associations with life satisfaction through eHealth literacy followed by (a) lower psychosocial loss, (b) more positive appraisals of physical aging, and (c) greater psychological growth.*

## 2. Materials and Methods

### 2.1. Study Design and Participants

This cross-sectional study used data from respondents aged 60 years or older in a community-based digital literacy survey of adults aged 18 years or older in S City, China. One community school in the central urban area and one in the suburban area were purposively selected from existing monitoring sites. The broader survey set a recruitment target of 750 respondents per area, comprising 250 in each of three age groups (18–29, 30–59, and ≥60 years), and sought an approximately balanced gender distribution in each area’s overall sample. These recruitment targets were set to facilitate comparisons across areas and age groups. Recruitment used convenience sampling and participant referrals. Community-school staff invited students through class messaging groups and organized their participation. Residents’ committees recruited community residents through community activities they organized. Participants could also share the questionnaire link with others. Participation was voluntary and required the ability to understand the questionnaire and communicate responses. Recruitment was coordinated locally and extended through link sharing. A complete record of all individuals approached or invited was unavailable, so a response rate could not be calculated.

The broader survey received 1487 questionnaires. Initial data cleaning excluded questionnaires with recorded completion times of 4 min or less, leaving 1325 respondents, of whom 604 were aged ≥60 years. The primary analysis included all 604 age-eligible respondents. Following data collection, responses were screened for invariant responding across six multi-item measures comprising 29 items: digital activity frequency (4 items), eHEALS (8 items), the three AAQ subscales—psychosocial loss, physical change, and psychological growth (4 items each)—and SWLS (5 items). This screening identified 118 respondents (19.5%) who selected the same response option for every item within each of all six measures. Each AAQ subscale was assessed separately, and the repeated response option could differ between measures. To assess the sensitivity of the findings to these response patterns, the analyses were repeated after excluding these 118 respondents, yielding a sensitivity-analysis sample of 486. Demographic comparisons between the 486 retained and 118 excluded respondents are presented in Supplementary Table S1. Results comparing the primary analysis (*N* = 604) and sensitivity analysis (*N* = 486) are reported in Section 3.3 and Supplementary Table S2.

The primary analytic sample included 427 women (70.7%) and 177 men (29.3%). Of the participants, 281 (46.5%) were aged 60–69 years, 257 (42.5%) were aged 70–79 years, and 66 (10.9%) were aged 80 years or older. The sample included 303 participants (50.2%) from the central urban area and 301 (49.8%) from the suburban area. Additional sample characteristics are presented in Table 1.

### 2.2. Measures

#### 2.2.1. Digital Activity Frequency

Digital activity frequency was assessed using four items adapted from the China Family Panel Studies (CFPS) 2018 questionnaire [33], covering online learning, work-related activities, entertainment, and social interaction. These four domains correspond to the activity indicators described and modeled by Wu et al. [34]. The commercial-activity frequency item in the CFPS questionnaire was not collected. The social-interaction example was changed from posting on Weibo to posting on WeChat Moments, and the work item referred broadly to work-related online activities, omitting the original clarification about an employer’s internal network. All participants completed all four items. Responses were coded from 1 (never) to 7 (almost daily), reversing the original CFPS coding direction. The four item scores were averaged to create an observed summary index ranging from 1 to 7, with higher scores indicating more frequent reported activity on average across the four domains. In the present sample, Cronbach’s α was 0.894. Confirmatory factor analysis (CFA) was conducted to further examine the relationships among the four items. Model specifications and detailed results are presented in Supplementary Table S4.

#### 2.2.2. eHealth Literacy

Perceived eHealth literacy was assessed using the eight-item eHealth Literacy Scale (eHEALS) [10] and the Chinese version developed by Chang and Schulz [35]. The items assessed participants’ perceived knowledge, confidence, and skills in finding, evaluating, and using online health information. Each item was rated from 1 (strongly disagree) to 5 (strongly agree). Item scores were summed, producing a total score ranging from 8 to 40, with higher scores indicating greater perceived eHealth literacy. In the present sample, Cronbach’s α was 0.984.

#### 2.2.3. Attitudes Toward Aging

Attitudes toward aging were assessed using the 12-item brief version of the Attitudes to Ageing Questionnaire for older Chinese adults (AAQ-BC) [36], which was developed from the original AAQ [22]. The instrument comprises three four-item subscales: psychosocial loss, physical change, and psychological growth. Items were rated from 1 (strongly disagree) to 5 (strongly agree) and summed within each subscale, producing scores ranging from 4 to 20. Psychosocial-loss items were not reverse-scored; therefore, higher scores indicated greater perceived loss. Higher scores for physical change and psychological growth indicated more positive appraisals. The physical-change subscale assesses subjective appraisals of physical aging rather than objective health or functional status. In the present sample, Cronbach’s α was 0.884 for psychosocial loss, 0.898 for physical change, and 0.845 for psychological growth.

#### 2.2.4. Life Satisfaction

Life satisfaction was assessed using the five-item Satisfaction With Life Scale (SWLS) [1] and the Chinese version validated by Xiong and Xu [37]. Items were rated on a seven-point scale ranging from 1 (strongly disagree) to 7 (strongly agree). Item scores were summed to produce a total score ranging from 5 to 35, with higher scores indicating greater global life satisfaction. In the present sample, Cronbach’s α was 0.943.

#### 2.2.5. Covariates

Covariates included gender, education, age category, living arrangement, and community location. Gender was recorded as woman or man. Education was categorized as junior high school or below, high or technical secondary school, vocational college, and bachelor’s degree or above. Age was categorized as 60–69, 70–79, and 80 years or older. Living arrangement was categorized as living alone, living with a spouse only, living with children or parents, and other arrangements. Community location identified the central urban or suburban recruitment area. Categorical covariates were represented by indicator variables, with women, junior high school or below, 60–69 years, living with a spouse only, and the suburban area as the reference categories.

### 2.3. Procedure

The questionnaire was hosted on Wenjuanxing. Community-school staff organized group completion by students in school meeting rooms, while residents’ committees organized sessions for community residents at neighborhood centers. Participants primarily accessed the questionnaire by scanning a QR code. A printed copy of the online questionnaire, with identical item wording, sequence, response options, and instructions, was available as a reading aid. When needed, staff read the questions aloud and entered participants’ stated responses directly into Wenjuanxing during completion. All responses were submitted through Wenjuanxing, which required responses to applicable items before submission. The use and extent of staff assistance were not separately recorded. Participants could also share the questionnaire link with family members or peers for independent completion. The research team monitored completed questionnaires and provided daily recruitment-count updates to the participating organizations. Recruitment ended at the close of the prespecified survey period. Participation was voluntary and anonymous. Written or electronic informed consent was obtained before questionnaire completion, and each participant received a small gift valued at approximately RMB 10. The study was approved by the Academic Ethics and Morality Committee of Shanghai Normal University (approval no. 094/2025).

### 2.4. Analytic Strategy

Analyses were conducted in Python 3.12 using statsmodels 0.14.6. The dataset used for the present analyses contained no missing values for the study variables or covariates. Means, standard deviations, Cronbach’s α coefficients, and Pearson correlations were calculated. Score distributions and bivariate relationships were assessed graphically, and the influence of individual observations was examined using leave-one-out diagnostics. Spearman correlations were calculated as a sensitivity analysis. The six study variables were standardized to *z* scores within each analytic sample, so that the path coefficients and indirect effects were expressed in standard-deviation units.

Regression-based mediation analysis was conducted using a series of ordinary least squares (OLS) equations fitted to observed composite scores. eHealth literacy was regressed on digital activity frequency, and each aging-attitude dimension was regressed on digital activity frequency and eHealth literacy. Life satisfaction was then regressed on digital activity frequency, eHealth literacy, and all three aging-attitude dimensions simultaneously. A separate model excluding the mediators estimated the total association between digital activity frequency and life satisfaction. All models adjusted for gender, education, age category, living arrangement, and community location. Regression inference used HC3 heteroskedasticity-robust standard errors. *R*^2^ was calculated for each model. Variance inflation factors were examined to assess multicollinearity in the final regression model.

Specific indirect associations were calculated as products of the regression coefficients along each proposed pathway, and the total indirect association was their sum. For each of 5000 bootstrap resamples, participants were sampled with replacement, the same resampled records were used across all regression equations, and all equations were refitted to recalculate the indirect associations. We obtained 95% confidence intervals from the resulting bootstrap distributions.

Two sensitivity analyses examined the robustness of the findings. First, the models were re-estimated after excluding the 118 respondents identified by the invariant-response screen, leaving 486 respondents. Second, the models were re-estimated in the primary sample (*N* = 604) after omitting the work-related activity item; digital activity frequency was calculated as the mean of online learning, entertainment, and social interaction. Both analyses used the same covariates and regression structure as the primary analysis. The total and direct associations and the specific and total indirect associations were compared across analyses.

## 3. Results

### 3.1. Descriptive Statistics and Correlations

Means, standard deviations, and Pearson correlations are presented in Table 2. Digital activity frequency was positively correlated with eHealth literacy, physical change, psychological growth, and life satisfaction (*r*s = 0.393–0.624, all *p*s < 0.001); its correlation with psychosocial loss was not statistically significant (*r* = 0.064, *p* = 0.117). eHealth literacy was positively correlated with all three aging-attitude dimensions and life satisfaction (*r*s = 0.161–0.564, all *p*s < 0.001). All three aging-attitude dimensions were positively correlated with life satisfaction (*r*s = 0.185–0.508, all *p*s < 0.001).

Spearman correlations showed the same directions as Pearson correlations across all 15 variable pairs, with a maximum absolute coefficient difference of 0.073. Statistical significance was consistent for 14 pairs; the correlation between digital activity frequency and psychosocial loss reached statistical significance only in the Spearman analysis (ρ = 0.081, *p* = 0.046). Some bivariate relationships showed curvature, particularly that between psychosocial loss and life satisfaction. Item-level descriptive statistics and correlations of the four activity components with perceived eHealth literacy and life satisfaction are presented in Supplementary Table S3.

### 3.2. Mediation Analysis

In the model without the mediators, digital activity frequency was positively associated with life satisfaction, β = 0.518, 95% CI [0.425, 0.612], *p* < 0.001. The path estimates for the mediation model are presented in Table 3. Digital activity frequency was positively associated with eHealth literacy, β = 0.589, *p* < 0.001.

Digital activity frequency was positively associated with physical change (β = 0.116, *p* = 0.026) and psychological growth (β = 0.133, *p* = 0.006), while its association with psychosocial loss was not statistically significant (β = −0.030, *p* = 0.601). eHealth literacy was positively associated with all three aging-attitude dimensions: psychosocial loss (β = 0.210, *p* < 0.001), physical change (β = 0.473, *p* < 0.001), and psychological growth (β = 0.507, *p* < 0.001).

**Figure 1:**
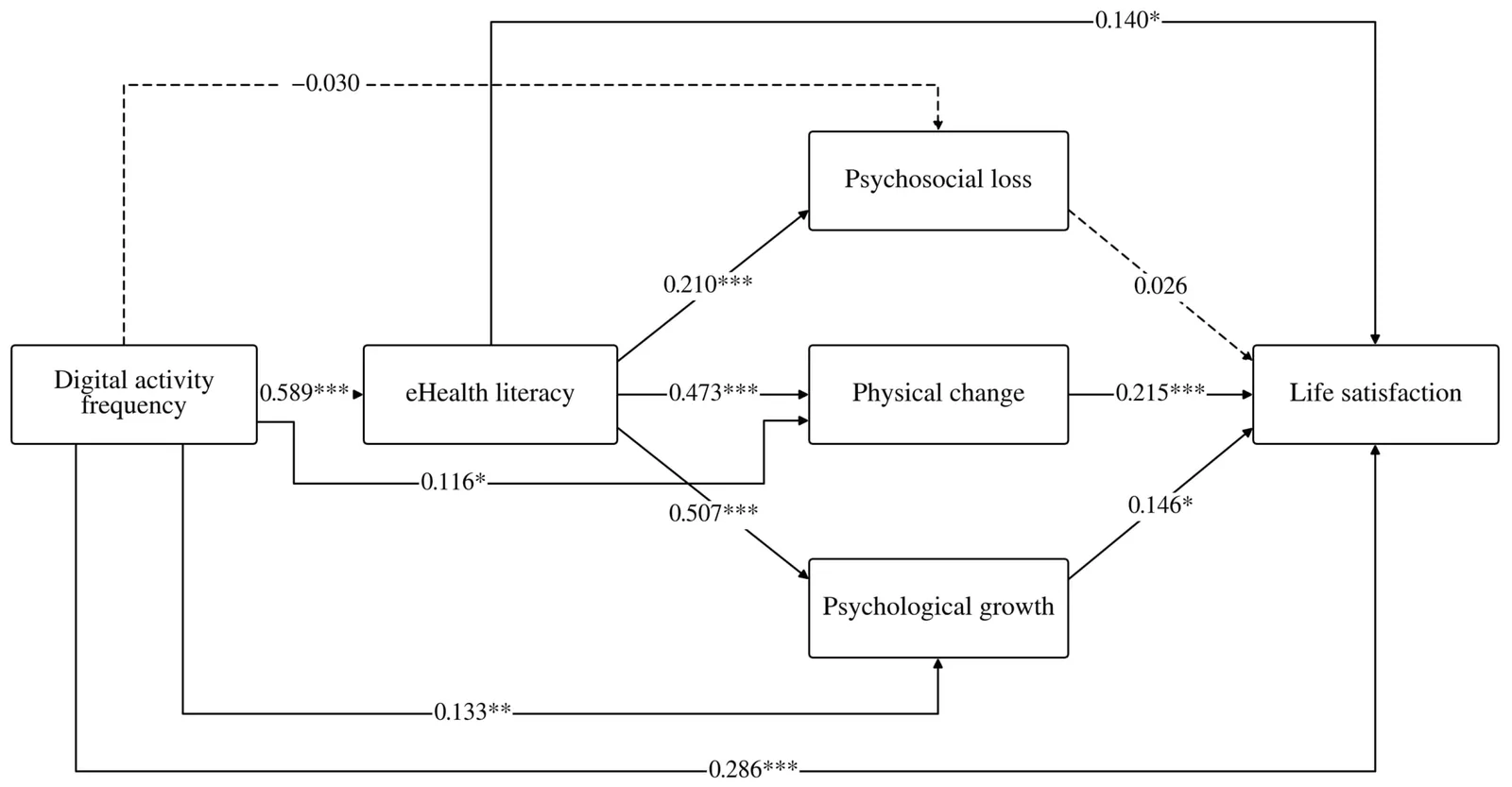
Integrated regression-based mediation model (*N* = 604). Coefficients are standardized regression estimates. Solid and dashed arrows indicate statistically significant and nonsignificant regression paths, respectively. All regression equations adjusted for gender, education, age category, living arrangement, and community location; covariates are omitted from the diagram for clarity. * *p* < 0.05; ** *p* < 0.01; *** *p* < 0.001.

When eHealth literacy and the three dimensions of attitudes toward aging were included in the same model, digital activity frequency remained positively associated with life satisfaction, β = 0.286, *p* < 0.001. eHealth literacy (β = 0.140, *p* = 0.028), physical change (β = 0.215, *p* < 0.001), and psychological growth (β = 0.146, *p* = 0.018) were also positively associated with life satisfaction. Psychosocial loss was not significantly associated with life satisfaction, β = 0.026, *p* = 0.432. The model accounted for 43.1% of the variance in life satisfaction. VIFs ranged from 1.08 to 2.25 in the final life satisfaction model, indicating no substantial multicollinearity. Figure 1 presents the integrated regression-based mediation model with standardized coefficients.

Table 4 presents the total and specific indirect associations. The total indirect association between digital activity frequency and life satisfaction was statistically significant, estimate = 0.233, 95% bootstrap CI [0.157, 0.316]. Five of the seven specific indirect associations were statistically significant. The indirect association through eHealth literacy alone was significant, estimate = 0.082, 95% bootstrap CI [0.013, 0.153], supporting H1. Neither indirect association involving psychosocial loss was significant. The indirect association through physical change alone (estimate = 0.025, 95% bootstrap CI [0.003, 0.058]) and the serial indirect association through eHealth literacy followed by physical change (estimate = 0.060, 95% bootstrap CI [0.028, 0.096]) were both statistically significant. The indirect association through psychological growth alone, estimate = 0.020, 95% bootstrap CI [0.003, 0.044], and the serial indirect association through eHealth literacy followed by psychological growth, estimate = 0.044, 95% bootstrap CI [0.010, 0.082], were both significant. H2 was therefore supported for the serial pathways through physical change and psychological growth, but not for the pathway through psychosocial loss.

### 3.3. Sensitivity Analyses

Results of the two sensitivity analyses are presented in Supplementary Table S2. After excluding the 118 respondents identified by the invariant-response screen, the total indirect association in the remaining 486 respondents was 0.209, 95% bootstrap CI [0.136, 0.287]. After omitting the work-related activity item in the primary sample (*N* = 604), the total indirect association was 0.212, 95% bootstrap CI [0.140, 0.293]. In both sensitivity analyses, the total and direct associations remained positive and statistically supported. The indirect association through eHealth literacy alone and the serial indirect associations through eHealth literacy followed by physical change or psychological growth also remained positive, with all corresponding 95% bootstrap confidence intervals excluding zero. The indirect associations through physical change alone and psychological growth alone were statistically supported in the primary analysis, but their confidence intervals included zero in both sensitivity analyses. Neither indirect pathway involving psychosocial loss was statistically supported in the primary or sensitivity analyses. The adjusted association between eHealth literacy and psychosocial loss was not statistically significant in the response-pattern sensitivity analysis (*N* = 486; β = 0.102, *p* = 0.134).

## 4. Discussion

This study examined indirect associations through perceived eHealth literacy and three dimensions of attitudes toward aging in the association between digital activity frequency and life satisfaction among older adults. More frequent digital activity was associated with greater life satisfaction, both directly and indirectly through eHealth literacy. Positive serial indirect associations were also found through eHealth literacy followed by physical change or psychological growth. Indirect associations through physical change alone and psychological growth alone were also observed, but neither was statistically supported in either sensitivity analysis. Neither pathway involving psychosocial loss was statistically supported. These findings highlight eHealth literacy and specific aging-related appraisals as potential links between older adults’ digital activity frequency and life satisfaction.

### 4.1. Digital Activity Frequency, eHealth Literacy, and Life Satisfaction

Digital activity frequency remained positively associated with life satisfaction after eHealth literacy and attitudes toward aging were included in the model, suggesting that additional pathways may be relevant. Digital communication may help older adults maintain valued relationships, while online learning and leisure may provide enjoyment and opportunities to pursue personal interests [38,39]. These experiences may relate to life satisfaction through social connectedness and fulfillment in everyday life, alongside the health-related pathway examined here.

A positive indirect association was also observed through eHealth literacy. Previous research has linked internet use to general digital literacy among older adults [40] and identified eHealth literacy as a mediator between general digital literacy and quality of life [19]. The present findings extend this work by identifying an indirect association between routine digital activity frequency and life satisfaction through perceived competence in finding, evaluating, and using online health information. Although empowerment was not directly assessed, Health Empowerment Theory offers a perspective for understanding how this competence may function as a personal resource in everyday health decisions [16]. Feeling able to use online health information may help older adults navigate health-related choices and feel more in control of managing their health, potentially contributing to greater life satisfaction.

### 4.2. The Pathway Through Physical Change

The physical change dimension captures older adults’ subjective evaluations of physical aging rather than objective physical health or functional status. Digital activity frequency showed a positive indirect association with life satisfaction through physical change alone. One possible explanation is that more frequent engagement in these digital activities may offer opportunities to pursue interests and maintain everyday involvement as physical circumstances change [38]. Being able to continue learning, communicating, and pursuing valued activities online may help older adults view physical aging as compatible with continued participation in everyday life. Such experiences may support more favorable appraisals of physical aging, which may, in turn, be associated with greater life satisfaction. However, the physical-change-only indirect association was not statistically supported in either sensitivity analysis and should therefore be interpreted cautiously.

A positive serial indirect association was also observed through eHealth literacy followed by physical change. This pathway suggests a complementary interpretation centered on perceived competence in using health information. Experience with routine digital activities may be accompanied by greater confidence in finding, evaluating, and using online health information. Feeling able to use this information may help older adults identify ways to respond to bodily changes and adapt everyday activities to changing health needs. Previous research has linked eHealth literacy to health-promoting behaviors and self-care [9,27]. Related evidence on health adaptation comes from Wurm et al. [41], who linked the use of selection, optimization, and compensation strategies following serious health events to better subsequent self-rated health and life satisfaction; negative perceptions of physical aging were associated with a weaker adaptive response. Within the present pathway, perceived competence in using health information may similarly represent a resource for understanding bodily changes and identifying possible responses. This sense that physical changes can be understood and addressed may support more favorable appraisals of physical aging and, in turn, greater life satisfaction.

### 4.3. The Pathway Through Psychological Growth

A small positive indirect association between digital activity frequency and life satisfaction was observed through psychological growth alone. More frequent engagement in these digital activities may provide opportunities to encounter new ideas, pursue interests, exchange experiences, and apply accumulated knowledge. These opportunities may help older adults perceive that later life still offers possibilities for development and that their experience remains valuable. Related evidence comes from a single-group pretest–posttest study in which older adults reported more positive attitudes toward their own aging after participating in digital literacy training [42]. In this interpretation, the opportunities accompanying digital activity may support perceptions of psychological growth, helping older adults regard their current stage of life as worthwhile and evaluate their lives more favorably. However, this indirect association was not statistically supported in either sensitivity analysis, and its small magnitude also warrants caution about its substantive importance.

The more consistent serial association points to perceived eHealth literacy as a potentially relevant resource within this process. Health Empowerment Theory emphasizes recognizing and drawing on personal resources to participate in health decisions and pursue health goals [16]. From this perspective, opportunities to engage with information during routine digital activities may acquire personal significance when accompanied by confidence in understanding, evaluating, and applying health information. Feeling able to address personally relevant health questions and draw on accumulated experience may affirm older adults’ continuing capacity to learn and exercise judgment. Such experiences may support a view of aging that recognizes the enduring value of experience and the possibility of further growth. Age-related beliefs can become part of how individuals understand themselves and their possibilities in later life [23]. Viewing aging as involving valuable experience and continued growth may therefore help older adults recognize gains in their current lives and regard this stage of life as worthwhile. This appreciation of their present stage of life may support a more favorable evaluation of life as a whole, consistent with evidence linking positive attitudes toward aging to life satisfaction and quality of life [24,32].

### 4.4. The Pathway Through Psychosocial Loss

Perceived eHealth literacy was positively associated with psychosocial loss, and this association remained positive after adjustment for digital activity frequency and covariates. Psychosocial loss concerns perceived loneliness, social exclusion, and reduced involvement [22,43]. One possible contributor to the association with perceived eHealth literacy is that health concerns or unmet support needs may encourage engagement with online health information while also accompanying stronger perceptions of loss. Related research during the COVID-19 pandemic found that searching online for health information was associated with greater loneliness among middle-aged and older adults, particularly those with limiting illnesses [44]. This provides a context for considering how health and social circumstances may shape the relationship between perceived health-information competence and psychosocial loss. However, the positive association between perceived eHealth literacy and psychosocial loss was not statistically supported in the response-pattern sensitivity analysis involving 486 respondents and therefore warrants cautious interpretation.

The positive association between psychosocial loss and life satisfaction differed from previous findings linking greater psychosocial loss to lower life satisfaction among Chinese older adults [28]. One possible interpretation concerns how older adults evaluate their current lives in relation to their expectations. Perceived age-related losses may carry different implications for life satisfaction depending on the standards against which current circumstances are judged. As circumstances change, older adults may adjust their expectations and reconsider the importance of different life domains. Longitudinal research suggests that such adjustments in personal comparison standards can help preserve favorable self-evaluations despite functional losses [45]. These processes may help explain how perceptions of psychosocial loss can accompany a favorable overall evaluation of life. In the present study, however, the association weakened and was no longer statistically significant after adjustment for digital activity frequency, perceived eHealth literacy, the other aging-attitude dimensions, and covariates. It was also not statistically supported in the response-pattern sensitivity analysis, indicating that this interpretation remains tentative. Neither the indirect association through psychosocial loss alone nor the serial indirect association through perceived eHealth literacy and psychosocial loss was statistically supported.

More frequent digital activity may provide opportunities for contact, but whether these encounters offer emotional support, meaningful interaction, and a sense of belonging may depend on the quality of the interactions. Similarly, perceived eHealth literacy may support participation in health decisions without necessarily corresponding to changes in relationship quality or experiences of social acceptance. Reviews of technology interventions also suggest variation in social outcomes. Shah et al. [46] found no clear evidence that digital technology interventions reduced loneliness, whereas Balki et al. [47] identified potential improvements in social connectedness but noted the limited certainty of the evidence. These findings suggest that opportunities for digital contact and perceived health-information competence may not consistently correspond to the relational experiences relevant to psychosocial loss, offering one possible explanation for the unsupported indirect pathways. Neither indirect pathway involving psychosocial loss was statistically supported in either sensitivity analysis. Future research could examine interaction quality, emotional support, and belonging to clarify how digital activity relates to psychosocial loss and life satisfaction.

### 4.5. Theoretical Implications

These findings highlight the value of considering perceived health-information competence and aging-related appraisals together when interpreting the association between digital activity frequency and life satisfaction. Health Empowerment Theory emphasizes recognizing and drawing on personal resources, offering a perspective on eHealth literacy as a resource for health-related decisions and goal pursuit [16]. Stereotype Embodiment Theory addresses how age-related beliefs become incorporated into self-perceptions and relate to experiences through psychological and behavioral processes [23]. Together, these perspectives direct attention to both health-related resources and individuals’ interpretations of their own aging. The supported serial indirect associations through eHealth literacy followed by physical change or psychological growth are consistent with an interpretation in which perceived health-information competence is associated with aging-related appraisals that, in turn, relate to life satisfaction.

A further implication is the importance of distinguishing the content of these appraisals. The supported serial pathways involving physical change and psychological growth, alongside the unsupported pathway involving psychosocial loss, suggest that an overall measure of attitudes toward aging could obscure dimension-specific patterns. This distinction is consistent with a lifespan perspective on views of aging, which recognizes that individuals may evaluate aging differently across life domains and perceive gains and losses concurrently [25]. Examining these dimensions separately therefore provides a more differentiated account of the aging-related appraisals involved. Empowerment processes and stereotype internalization were not directly assessed in this study; future research measuring these processes could clarify the theoretical interpretation of the observed associations.

### 4.6. Practical Implications

The findings identify perceived competence in using health information, appraisals of physical aging, and perceptions of psychological growth as potential targets for future community-based digital health support. Community health professionals and educators could explore combining practice in finding and evaluating information about everyday health concerns with discussions of bodily changes, adaptation, and continued learning. For example, participants could compare health-information sources and discuss how accumulated experience can help them interpret new information and respond to health needs. Activities could be adapted to participants’ digital experience and resources through step-by-step demonstrations and individual assistance for less experienced users, and access to devices and internet connections at community venues for those with limited resources. Easy-to-read materials and face-to-face discussion could further support participation across different levels of digital familiarity.

The effects of such support on physical-aging appraisals, psychological growth, and life satisfaction remain to be tested. Future evaluations could assess confidence in using health information alongside actual task performance, complementing the perceived eHealth literacy measure used here. The broad frequency-based activity items also leave the appropriate content, frequency, and modes of interaction for future investigation. Given the unsupported psychosocial-loss pathway, support addressing loneliness, exclusion, or belonging warrants separate investigation that considers interpersonal interaction and social support.

### 4.7. Limitations

This study has several limitations. First, the cross-sectional design does not establish temporal sequence or causal mediation. The estimated indirect associations are statistical associations under a theoretically specified ordering. Alternative or reciprocal directions remain possible: greater life satisfaction could encourage more frequent digital activity, while more favorable aging appraisals could strengthen confidence in using digital health information or encourage digital engagement. Longitudinal studies with repeated assessments of all focal variables are needed to examine the proposed ordering and these alternative directions.

Although the analyses adjusted for demographic and residential characteristics, the broader survey did not collect independent measures of physical health, multimorbidity, functional status, cognitive status, income, wealth, or social support. Residual confounding by health, economic, and social circumstances, including social support, remains possible. These factors may act as common determinants of the study variables and account for part of the estimated direct and indirect associations. This concern is particularly relevant to the physical-change pathway: underlying health and functional limitations could be related simultaneously to digital activity frequency, perceived eHealth literacy, appraisals of physical aging, and life satisfaction, potentially accounting for part of the estimated indirect associations. The AAQ physical-change subscale measures subjective aging appraisals and does not substitute for an independent assessment of health or functioning. Future studies should also assess individual care complexity, including care needs and vulnerability that conventional clinical measures may not fully capture [48]. Such differences may be relevant to perceived eHealth literacy and its association with life satisfaction. The magnitude and direction of any bias arising from these unmeasured characteristics cannot be determined from the present data.

Second, nonprobability recruitment through community schools, residents’ committees, and participant referrals in two areas of one city may have introduced selection bias and limits the generalizability of the findings. The absence of a complete invitation count also prevented calculation of a response rate and assessment of nonresponse bias. Digital familiarity may have influenced participation, while staff assistance may have affected how questions were understood and answered. The use and extent of assistance were not separately recorded, preventing examination of differences between independent and staff-assisted completion. Future studies should include older adults from a wider range of settings and levels of digital familiarity and record the assistance provided during questionnaire completion.

Third, all focal constructs were assessed by self-report within the same survey. Common-method variance arising from shared response tendencies, together with recall bias, may have influenced the observed associations. In particular, eHEALS assesses perceived eHealth literacy rather than performance on health information tasks. Its exceptionally high internal consistency may also reflect overlapping item content. The digital activity index summarizes frequency across four selected activities; equal scores may represent different activity profiles. It does not capture all dimensions of digital participation, the content or quality of activity, or whether “never” reflects non-use or non-applicability of work-related activity. Future research should examine activity-specific associations and combine self-report measures with performance-based assessments of eHealth literacy.

## 5. Conclusions

In this cross-sectional study, more frequent digital activity was associated with greater life satisfaction. Indirect associations were observed through eHealth literacy alone and through eHealth literacy followed by either physical change or psychological growth. Indirect associations through physical change alone and psychological growth alone were also observed, but neither was statistically supported in either sensitivity analysis. Neither indirect pathway involving psychosocial loss was statistically supported. The direct association between digital activity frequency and life satisfaction remained after inclusion of the proposed mediators, indicating that these variables provided a partial statistical account of the association. These findings are consistent with HET- and SET-informed interpretations and highlight the value of distinguishing specific aging-related appraisals. Perceived eHealth literacy, appraisals of physical aging, and perceptions of psychological growth represent potential targets for future community-based support development and testing. Longitudinal and intervention studies are needed to examine the temporal ordering and potential causal relationships underlying these associations.

## Figures and Tables

**Table 1:** Characteristics of the primary analytic sample (*N* = 604).

Characteristic	*n*	%
Gender: Women	427	70.7
Gender: Men	177	29.3
Age category: 60–69 years	281	46.5
Age category: 70–79 years	257	42.5
Age category: ≥80 years	66	10.9
Education: Junior high school or below	253	41.9
Education: High/technical secondary school	206	34.1
Education: Vocational college	96	15.9
Education: Bachelor’s degree or above	49	8.1
Living arrangement: Living alone	64	10.6
Living arrangement: Living with a spouse only	329	54.5
Living arrangement: Living with children or parents	95	15.7
Living arrangement: Other arrangements	116	19.2
Community location: Suburban	301	49.8
Community location: Central urban	303	50.2

Note. Percentages may not sum to 100 because of rounding.

**Table 2:** Means, standard deviations, and Pearson correlations among the study variables (*N* = 604).

Variable	*M*	*SD*	1	2	3	4	5	6
1. Digital activity frequency	4.71	1.86	1.00					
2. eHealth literacy	28.46	8.87	0.624 ***	1.00				
3. Psychosocial loss	12.32	4.20	0.064	0.161 ***	1.00			
4. Physical change	14.54	3.82	0.393 ***	0.518 ***	0.375 ***	1.00		
5. Psychological growth	13.81	3.79	0.417 ***	0.564 ***	0.297 ***	0.685 ***	1.00	
6. Life satisfaction	26.31	6.61	0.525 ***	0.514 ***	0.185 ***	0.508 ***	0.503 ***	1.00

Note. Higher psychosocial loss scores indicate greater perceived loss; higher physical change and psychological growth scores indicate more positive appraisals; *** *p* < 0.001, two-tailed.

**Table 3:** Standardized regression coefficients for the mediation model (*N* = 604).

Outcome	Predictor	β	SE	95% CI	*p*	*R* ^2^
eHealth literacy	Digital activity frequency	0.589	0.041	[0.508, 0.669]	<0.001	0.408
Psychosocial loss	Digital activity frequency	−0.030	0.057	[−0.141, 0.081]	0.601	0.053
	eHealth literacy	0.210	0.059	[0.094, 0.325]	<0.001	
Physical change	Digital activity frequency	0.116	0.052	[0.014, 0.217]	0.026	0.297
	eHealth literacy	0.473	0.058	[0.359, 0.588]	<0.001	
Psychological growth	Digital activity frequency	0.133	0.049	[0.038, 0.229]	0.006	0.348
	eHealth literacy	0.507	0.055	[0.400, 0.615]	<0.001	
Life satisfaction	Digital activity frequency	0.286	0.060	[0.168, 0.403]	<0.001	0.431
	eHealth literacy	0.140	0.064	[0.015, 0.265]	0.028	
	Psychosocial loss	0.026	0.033	[−0.039, 0.090]	0.432	
	Physical change	0.215	0.060	[0.098, 0.332]	<0.001	
	Psychological growth	0.146	0.062	[0.025, 0.267]	0.018	

Note. All study variables shown in the table were standardized. All equations adjusted for gender, education, age category, living arrangement, and community location. Reference categories were women, junior high school or below, age 60–69 years, living with a spouse only, and the suburban area, respectively. SE = standard error. Regression standard errors and confidence intervals were based on HC3 heteroskedasticity-robust covariance estimates.

**Table 4:** Indirect associations between digital activity frequency and life satisfaction (*N* = 604).

Path	Standardized Estimate	Bootstrap SE	95% Bootstrap CI
**Single-mediator indirect associations**			
Digital activity frequency → eHealth literacy → Life satisfaction (H1)	0.082	0.036	[0.013, 0.153]
Digital activity frequency → Psychosocial loss → Life satisfaction	−0.001	0.003	[−0.007, 0.004]
Digital activity frequency → Physical change → Life satisfaction	0.025	0.014	[0.003, 0.058]
Digital activity frequency → Psychological growth → Life satisfaction	0.020	0.011	[0.003, 0.044]
**Serial indirect associations**			
Digital activity frequency → eHealth literacy → Psychosocial loss → Life satisfaction (H2(a))	0.003	0.004	[−0.005, 0.012]
Digital activity frequency → eHealth literacy → Physical change → Life satisfaction (H2(b))	0.060	0.017	[0.028, 0.096]
Digital activity frequency → eHealth literacy → Psychological growth → Life satisfaction (H2(c))	0.044	0.018	[0.010, 0.082]
**Total indirect association**	0.233	0.041	[0.157, 0.316]

Note. CI = confidence interval; SE = standard error. Estimates are standardized indirect associations. Percentile 95% bootstrap confidence intervals were based on 5000 participant-level resamples. An indirect association was considered statistically significant when its confidence interval excluded zero. All equations adjusted for gender, education, age category, living arrangement, and community location, using the reference categories specified in Table 3.

## Data Availability

The data supporting the findings of this study are available from the corresponding author upon reasonable request. The full dataset is not publicly available because it also contains data for other studies that have not yet been published.

## Supplementary Materials

The following supporting information can be downloaded at https://www.mdpi.com/article/10.3390/healthcare14213816/s1: Table S1. Sociodemographic characteristics by response-pattern screening status within the primary sample (*N* = 604); Table S2. Total, direct, and indirect associations in the primary and sensitivity analyses; Table S3. Item-level descriptive statistics and correlations with perceived eHealth literacy and life satisfaction (*N* = 604); Table S4. Original and modified one-factor CFA models for the four digital activity frequency items [49,50].
