# Supplementary material for: Digital Activity Frequency and Life Satisfaction Among Older Adults: Indirect Associations Through eHealth Literacy and Attitudes Toward Aging
Source: Healthcare (Basel). 2026 Nov 9;14(21):3816. doi: 10.3390/healthcare14213816 (PMC13762887; doi:10.3390/healthcare14213816)
Supplement: Supplementary file 1 [file healthcare-14-03816-s001.zip › healthcare-4618790-supplementary.pdf]

Supplementary Materials

Digital Activity Frequency and Life Satisfaction Among Older Adults: Indirect Associations Through eHealth Literacy and Attitudes Toward Aging

Table S1. Sociodemographic characteristics by response-pattern screening status within the primary sample (N = 604).

Characteristic	Retained for sensitivity analysis (N = 486), n (%)	Excluded for sensitivity analysis (N = 118), n (%)	χ^2 (df)	p	Cramer's V
Gender: Women	346 (71.2)	81 (68.6)	0.30 (1)	0.585	0.022
Men	140 (28.8)	37 (31.4)			
Age category: 60 – 69 years	232 (47.7)	49 (41.5)	2.49 (2)	0.288	0.064
70 – 79 years	205 (42.2)	52 (44.1)			
80 years or older	49 (10.1)	17 (14.4)			
Education: Junior high school or below	204 (42.0)	49 (41.5)	2.28 (3)	0.516	0.061
High/technical secondary school	160 (32.9)	46 (39.0)			
Vocational college	81 (16.7)	15 (12.7)			
Bachelor's degree or above	41 (8.4)	8 (6.8)			
Living arrangement: Living alone	52 (10.7)	12 (10.2)	0.38 (3)	0.945	0.025
Living with a spouse only	266 (54.7)	63 (53.4)			
Living with children or parents	77 (15.8)	18 (15.3)			
Other arrangements	91 (18.7)	25 (21.2)			
Community location: Suburban	239 (49.2)	62 (52.5)	0.43 (1)	0.512	0.027
Central urban	247 (50.8)	56 (47.5)			

Note. All 604 respondents were included in the primary analysis. The sensitivity analysis omitted 118 respondents who selected the same option for every item within each of all six multi-item measures; the selected option could differ between measures. The screen covered 29 items: digital activity frequency (4), eHEALS (8), the three AAQ subscales (4 each), and SWLS (5). Values are n (%), with within-group percentages. Group comparisons used two-sided Pearson chi-square tests without continuity correction.

Table S2. Total, direct, and indirect associations in the primary and sensitivity analyses

Association / indirect pathway	Primary analysis<br>N = 604	Response-pattern sensitivity analysis<br>N = 486	Work-related item omitted<br>N = 604
Total association	0.518<br>[0.425, 0.612]	0.407<br>[0.299, 0.514]	0.523<br>[0.427, 0.619]
Direct association	0.286<br>[0.168, 0.403]	0.198<br>[0.080, 0.316]	0.311<br>[0.196, 0.426]
DAF → EHL → LS	0.082<br>[0.013, 0.153]	0.086<br>[0.015, 0.154]	0.073<br>[0.008, 0.141]
DAF → PL → LS	−0.001<br>[−0.007, 0.004]	−0.001<br>[−0.011, 0.009]	−0.002<br>[−0.010, 0.003]
DAF → EHL → PL → LS	0.003<br>[−0.005, 0.012]	0.001<br>[−0.005, 0.006]	0.004<br>[−0.004, 0.013]
DAF → PC → LS	0.025<br>[0.003, 0.058]	0.024<br>[−0.001, 0.059]	0.017<br>[−0.003, 0.048]
DAF → EHL → PC → LS	0.060<br>[0.028, 0.096]	0.052<br>[0.025, 0.085]	0.060<br>[0.028, 0.096]
DAF → PG → LS	0.020<br>[0.003, 0.044]	0.017<br>[−0.000036, 0.042]	0.014<br>[−0.000104, 0.036]
DAF → EHL → PG → LS	0.044<br>[0.010, 0.082]	0.031<br>[0.003, 0.063]	0.045<br>[0.012, 0.083]
Total indirect association	0.233<br>[0.157, 0.316]	0.209<br>[0.136, 0.287]	0.212<br>[0.140, 0.293]

Note. Values are standardized estimates with 95% confidence intervals in brackets. Total and direct association intervals use HC3 robust standard errors; indirect association intervals use 5,000 participant-level percentile bootstrap resamples. All equations adjusted for gender, education, age category, living arrangement, and community location. The work-omission analysis used the mean of online learning, entertainment, and social interaction in the same 604 respondents. Confidence limits close to zero are shown to additional decimal places. DAF = digital activity frequency; EHL = perceived eHealth literacy; PL = psychosocial loss; PC = physical change; PG = psychological growth; LS = life satisfaction.

Table S3. Item-level descriptive statistics and correlations with perceived eHealth literacy and life satisfaction (N = 604).

Activity	M (SD)	Never, n (%)	Perceived eHealth literacy r [95% CI]	Life satisfaction r [95% CI]
Online learning	4.641 (2.118)	83 (13.74)	.585 [.530, .635]	.471 [.406, .531]
Work-related activities	4.136 (2.285)	143 (23.68)	.525 [.465, .581]	.385 [.315, .451]
Entertainment	4.947 (2.078)	72 (11.92)	.539 [.480, .594]	.482 [.418, .541]
Social interaction	5.131 (2.031)	62 (10.26)	.526 [.466, .582]	.502 [.440, .559]

Note. Each activity was rated from 1 (never) to 7 (almost daily). Correlations are unadjusted Pearson coefficients; 95% confidence intervals were calculated using Fisher's z transformation. All correlations shown had two-sided $p < .001$. M = mean; SD = standard deviation; CI = confidence interval.

Table S4. Original and modified one-factor CFA models for the four digital activity frequency items.

To examine relationships among the four digital activity items, we fitted a one-factor CFA model with uncorrelated residuals. During manuscript revision, we also explored a modified model allowing a residual covariance between online learning and work-related activities. Previous research has conceptualized learning and working as instrumental, goal-directed ICT use (Senkbeil, 2022), suggesting possible shared task-oriented features of these activities. This offers a content-based rationale for examining their residual association, consistent with evidence that similarities in item meaning can contribute to correlated residuals (Bandalos, 2021). The tables below present both models' fit indices and standardized loadings, together with the modified model's residual covariance. These analyses supplement the assessment of item relationships; regression analyses used the four-item mean as an observed composite of activity frequency.

A. Model fit of the original and modified models

Sample (N)	Model	x^2	df	p	CFI	TLI	RMSEA	RMSEA 90% CI	SRMR
Primary (604)	Original	69.53	2	< .001	.958	.874	.237	[.191, .286]	.042
Primary (604)	Modified	3.08	1	.079	.999	.992	.059	[.000, .138]	.006

B. Standardized factor loadings

Activity	Primary (N = 604) Original	Primary (N = 604) Modified
Online learning	0.813	0.789
Work-related activities	0.674	0.637
Entertainment	0.920	0.930
Social interaction	0.889	0.895

C. Learning–work residual association in the modified model

Sample (N)	Unstandardized residual covariance	Residual correlation
Primary (604)	0.812	0.355

Note. CFA = confirmatory factor analysis; CFI = comparative fit index; TLI = Tucker–Lewis index; RMSEA = root mean square error of approximation; SRMR = standardized root mean square residual; CI = confidence interval; df = degrees of freedom. Models used maximum likelihood estimation, treating the seven-category responses as numeric. Both models converged with positive residual variances. Both models were estimated in the primary analytic sample (N = 604).

References

Senkbeil, M. ICT-related variables as predictors of ICT literacy beyond intelligence and prior achievement. Education and In-formation Technologies, 2022, 27, 3595–3622. https://doi.org/10.1007/s10639-021-10759-x

Bandalos, D. L. Item meaning and order as causes of correlated residuals in confirmatory factor analysis. Structural Equation Modeling: A Multidisciplinary Journal, 2021, 28(6), 903–913. https://doi.org/10.1080/10705511.2021.1916395
